# A second monoclinic polymorph of di-μ-chlorido-bis­(chlorido{2-[(4-ethyl­phen­yl)imino­meth­yl]pyridine-κ^2^ *N*,*N*′}copper(II))

**DOI:** 10.1107/S1600536812026347

**Published:** 2012-06-16

**Authors:** Mehdi Khalaj, Saeed Dehghanpour, Ali Mahmoudi, Arash Khalaj, Alan J. Lough

**Affiliations:** a Department of Chemistry, Islamic Azad University, Buinzahra Branch, Qazvin, Iran; b Department of Chemistry, Alzahra University, Tehran, Iran; c Department of Chemistry, Islamic Azad University, Karaj Branch, Karaj, Iran; d Department of Chemistry, University of Toronto, 80 St. George St., Toronto, Ontario, Canada M5S 3H6

## Abstract

The title compound, [Cu_2_Cl_4_(C_14_H_14_N_2_)_2_], is a new polymorph of a previously reported compound [Dehghanpour *et al.* (2011 ▶). *Acta Cryst.* E**67**, m1296]. The current polymorph was obtained from an acetonitrile solution of the title compound. Like the first polymorph, it is monoclinic (space group *P*2_1_/*c*). The unique Cu^II^ ion in the title centrosymmetric dinuclear complex is in a distorted trigonal–bipyramidal coordination environment formed by the bis-­chelating *N*-heterocyclic ligand, two bridging Cl ligands and one terminal Cl ligand. In the crystal, weak C—H⋯Cl hydrogen bonds are observed in addition to π–π stacking inter­actions, with a centroid–centroid distance of 3.6597 (18) Å.

## Related literature

For the synthesis of the ligand, see: Dehghanpour *et al.* (2009 ▶). For background to diimine complexes and related structures, see: Dehghanpour *et al.* (2011 ▶); Salehzadeh *et al.* (2011 ▶). For an index of trigonality as a general descriptor of five-coord­inate complexes, see: Addison *et al.* (1984 ▶).
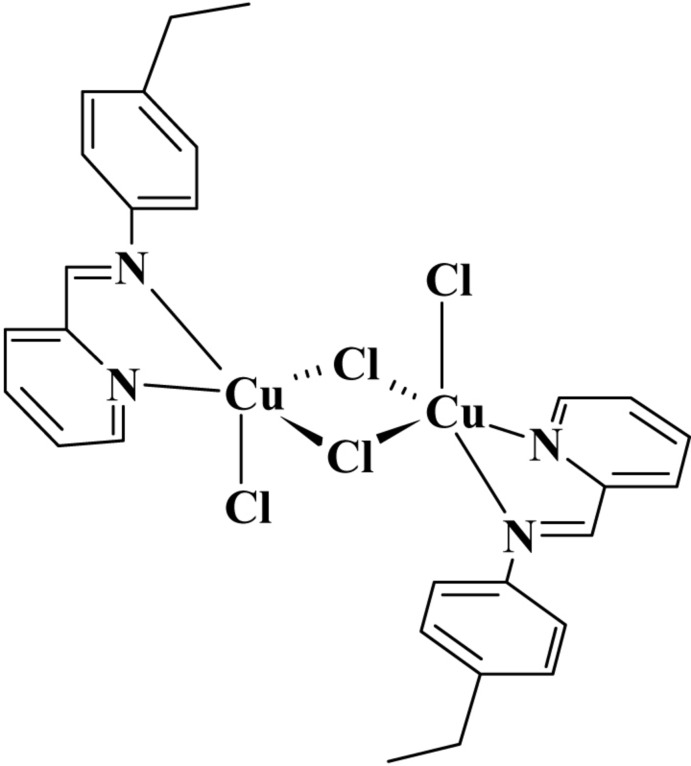


## Experimental

### 

#### Crystal data

[Cu_2_Cl_4_(C_14_H_14_N_2_)_2_]
*M* *_r_* = 689.42
Monoclinic, 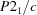

*a* = 7.8480 (4) Å
*b* = 13.7160 (6) Å
*c* = 14.4601 (7) Å
β = 113.924 (3)°
*V* = 1422.80 (12) Å^3^
*Z* = 2
Mo *K*α radiation
μ = 1.90 mm^−1^
*T* = 150 K
0.30 × 0.25 × 0.20 mm

#### Data collection

Nonius KappaCCD diffractometer
Absorption correction: multi-scan (*SORTAV*; Blessing, 1995 ▶) *T*_min_ = 0.581, *T*_max_ = 0.689
7862 measured reflections
3249 independent reflections
2399 reflections with *I* > 2σ(*I*)
*R*_int_ = 0.045

#### Refinement

*R*[*F* ^2^ > 2σ(*F* ^2^)] = 0.040
*wR*(*F* ^2^) = 0.102
*S* = 1.05
3249 reflections
173 parameters
H-atom parameters constrained
Δρ_max_ = 0.76 e Å^−3^
Δρ_min_ = −0.70 e Å^−3^

### 

Data collection: *COLLECT* (Nonius, 2002 ▶); cell refinement: *DENZO-SMN* (Otwinowski & Minor, 1997 ▶); data reduction: *DENZO-SMN*; program(s) used to solve structure: *SIR92* (Altomare *et al.*, 1994 ▶); program(s) used to refine structure: *SHELXTL* (Sheldrick, 2008 ▶); molecular graphics: *PLATON* (Spek, 2009 ▶) and *Mercury* (Macrae *et al.*, 2006) ▶; software used to prepare material for publication: *SHELXTL*.

## Supplementary Material

Crystal structure: contains datablock(s) I, global. DOI: 10.1107/S1600536812026347/fb2253sup1.cif

Structure factors: contains datablock(s) I. DOI: 10.1107/S1600536812026347/fb2253Isup2.hkl

Additional supplementary materials:  crystallographic information; 3D view; checkCIF report

## Figures and Tables

**Table 1:** Hydrogen-bond geometry (Å, °)

*D*—H⋯*A*	*D*—H	H⋯*A*	*D*⋯*A*	*D*—H⋯*A*
C1—H1*A*⋯Cl1	0.95	2.80	3.364 (3)	119
C2—H2*A*⋯Cl2^i^	0.95	2.76	3.445 (3)	130
C6—H6*A*⋯Cl2^ii^	0.95	2.62	3.506 (3)	155
C12—H12*A*⋯Cl2	0.95	2.80	3.450 (3)	126

Symmetry codes: (i) 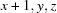; (ii) 
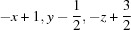
.

**Fig. 1.**
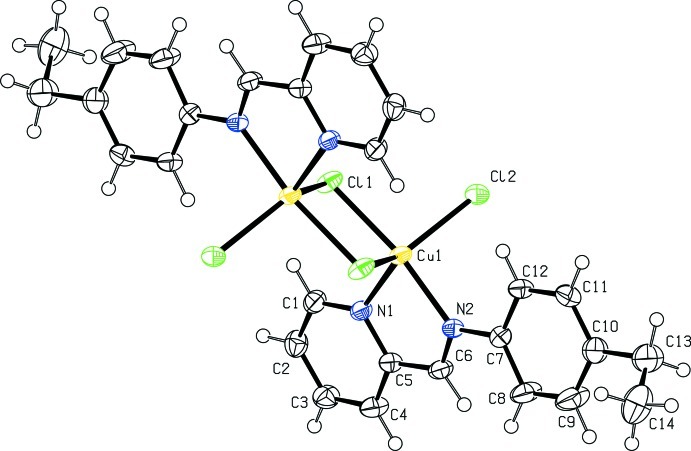
A view of the structure of the title complex, with displacement ellipsoids drawn at the 50% probability level [H atoms are represented as spheres of arbitrary radius]. Unlabelled atoms are related by the symmetry operator (-x+1, -y+1, -z+1).

**Fig. 2.**
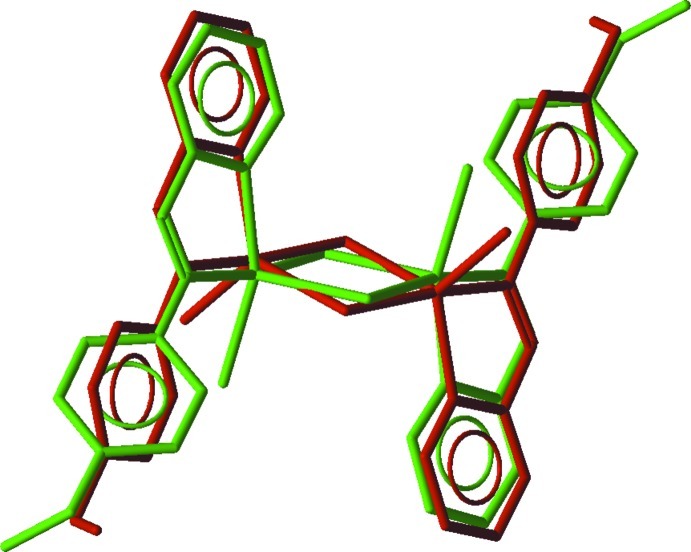
A comparison of both polymorphs. The title molecule is shown in red while that determined by Dehghanpour et al. (2011) in green (Mercury; Macrae et al., 2006).

## supplementary crystallographic information

### Comment

The crystal structure of a polymorph of the title compound has previously been reported (Dehghanpour *et al.*, 2011). In the course of our studies on the synthesis, structural and spectroscopic characterization of transition metal complexes with diimine ligands (Dehghanpour *et al.*, 2009; Salehzadeh *et al.*, 2011) a new polymorph of the title compound was obtained.

The title complex is shown in Fig. 1. The most significant structural difference between this structure and the polymorph (Dehghanpour *et al.*, 2011) is the coordination environment of the Cu^II^ ion. The structural index τ, (Addison *et al.*, 1984) which is a measure of trigonal distortion, is 0.75 for the title structure indicating a distorted trigonal-bipyramidal environment of Cu(II) for the title compound. The value of τ is 0.21 for the other polymorph with a distorted square-planar coordination enviroment. These differences are shown in Fig. 2.

The interplanar angles between the benzene and pyridine rings in the title structure is 12.40 (15)° whereas this angle is 43.02 (13)° in the polymorph determined by Dehghanpour *et al.* (2011).

In the crystal, weak C—H···Cl hydrogen bonds are observed in addition to π–π stacking interactions with a centroid to centroid distance of 3.6597 (18)Å for Cg1···Cg2^i^ (where Cg1 and Cg2 are centroids of the N1-C1-C5 and C7-C12 rings; symmetry code: 1+*x*, *y*, *z*).

### Experimental

The title complex was prepared by the reaction of CuCl_2_ (13.4 mg, 0.1 mmol) and (4-methylphenyl)pyridin-2-ylmethyleneamine (21.0 mg, 0.1) in 15 ml of acetonitrile at room temperature. The solution was allowed to stand at room temperature and orange block-shaped crystal of the title compound suitable for X-ray analysis precipitated within few days.

### Refinement

All the H atoms were located in the difference electron density map. Nevertheless, the H atoms were constrained and refined in the riding motion approximation: C_aryl_—H = 0.95, C_methylene_—H = 0.99, C_methyl_—H = 0.98 Å. *U*_iso_(H_aryl/methylene_) = 1.2 × *U*_eq_(C_carrier_) and *U*_iso_(H_methyl_) = 1.5 × *U*_eq_(C_carrier_).

### Crystal data

**Table:**

[Cu_2_Cl_4_(C_14_H_14_N_2_)_2_]	*F*(000) = 700
*M**_r_* = 689.42	*D*_x_ = 1.609 Mg m^−^^3^
Monoclinic, *P*2_1_/*c*	Mo *K*α radiation, λ = 0.71073 Å
Hall symbol: -P 2ybc	Cell parameters from 4440 reflections
*a* = 7.8480 (4) Å	θ = 2.6–27.5°
*b* = 13.7160 (6) Å	µ = 1.90 mm^−^^1^
*c* = 14.4601 (7) Å	*T* = 150 K
β = 113.924 (3)°	Block, orange
*V* = 1422.80 (12) Å^3^	0.30 × 0.25 × 0.20 mm
*Z* = 2	

### Data collection

**Table:**

Nonius KappaCCD diffractometer	3249 independent reflections
Radiation source: fine-focus sealed tube	2399 reflections with *I* > 2σ(*I*)
Graphite monochromator	*R*_int_ = 0.045
Detector resolution: 9 pixels mm^-1^	θ_max_ = 27.5°, θ_min_ = 2.8°
φ scans and ω scans with κ offsets	*h* = −10→10
Absorption correction: multi-scan (*SORTAV*; Blessing, 1995)	*k* = −17→15
*T*_min_ = 0.581, *T*_max_ = 0.689	*l* = −12→18
7862 measured reflections	

### Refinement

**Table:**

Refinement on *F*^2^	Primary atom site location: structure-invariant direct methods
Least-squares matrix: full	Secondary atom site location: difference Fourier map
*R*[*F*^2^ > 2σ(*F*^2^)] = 0.040	Hydrogen site location: difference Fourier map
*wR*(*F*^2^) = 0.102	H-atom parameters constrained
*S* = 1.05	*w* = 1/[σ^2^(*F*_o_^2^) + (0.0441*P*)^2^ + 0.8729*P*] where *P* = (*F*_o_^2^ + 2*F*_c_^2^)/3
3249 reflections	(Δ/σ)_max_ = 0.001
173 parameters	Δρ_max_ = 0.76 e Å^−^^3^
0 restraints	Δρ_min_ = −0.70 e Å^−^^3^
55 constraints	

### Special details

**Table:**

Geometry. All esds (except the esd in the dihedral angle between two l.s. planes) are estimated using the full covariance matrix. The cell esds are taken into account individually in the estimation of esds in distances, angles and torsion angles; correlations between esds in cell parameters are only used when they are defined by crystal symmetry. An approximate (isotropic) treatment of cell esds is used for estimating esds involving l.s. planes.
Refinement. Refinement of *F*^2^ against ALL reflections. The weighted *R*-factor *wR* and goodness of fit *S* are based on *F*^2^, conventional *R*-factors *R* are based on *F*, with *F* set to zero for negative *F*^2^. The threshold expression of *F*^2^ > σ(*F*^2^) is used only for calculating *R*-factors(gt) *etc*. and is not relevant to the choice of reflections for refinement. *R*-factors based on *F*^2^ are statistically about twice as large as those based on *F*, and *R*- factors based on ALL data will be even larger.

### Fractional atomic coordinates and isotropic or equivalent isotropic displacement parameters (Å^2^)

**Table:**

	*x*	*y*	*z*	*U*_iso_*/*U*_eq_	
Cu1	0.50129 (5)	0.45502 (2)	0.61249 (3)	0.02458 (13)	
Cl1	0.60970 (11)	0.59376 (5)	0.56397 (5)	0.0301 (2)	
Cl2	0.33826 (11)	0.53968 (5)	0.68632 (6)	0.03089 (19)	
N1	0.7670 (3)	0.40393 (17)	0.70183 (18)	0.0239 (5)	
N2	0.4320 (3)	0.32164 (16)	0.64881 (17)	0.0227 (5)	
C1	0.9333 (4)	0.4446 (2)	0.7264 (2)	0.0290 (7)	
H1A	0.9403	0.5066	0.6987	0.035*	
C2	1.0974 (4)	0.4000 (2)	0.7912 (2)	0.0329 (7)	
H2A	1.2139	0.4315	0.8077	0.040*	
C3	1.0897 (4)	0.3100 (2)	0.8311 (2)	0.0337 (7)	
H3A	1.2003	0.2784	0.8757	0.040*	
C4	0.9176 (4)	0.2662 (2)	0.8049 (2)	0.0342 (7)	
H4A	0.9079	0.2034	0.8301	0.041*	
C5	0.7598 (4)	0.3157 (2)	0.7415 (2)	0.0265 (6)	
C6	0.5738 (4)	0.2752 (2)	0.7108 (2)	0.0287 (7)	
H6A	0.5573	0.2140	0.7368	0.034*	
C7	0.2496 (4)	0.2788 (2)	0.6146 (2)	0.0262 (6)	
C8	0.2256 (5)	0.1806 (2)	0.6308 (3)	0.0442 (9)	
H8A	0.3310	0.1398	0.6634	0.053*	
C9	0.0487 (5)	0.1429 (3)	0.5996 (3)	0.0530 (11)	
H9A	0.0340	0.0758	0.6111	0.064*	
C10	−0.1092 (4)	0.1998 (2)	0.5515 (3)	0.0368 (8)	
C11	−0.0829 (4)	0.2962 (2)	0.5328 (2)	0.0302 (7)	
H11A	−0.1883	0.3364	0.4983	0.036*	
C12	0.0946 (4)	0.3355 (2)	0.5634 (2)	0.0279 (7)	
H12A	0.1094	0.4018	0.5491	0.033*	
C13	−0.3033 (5)	0.1564 (3)	0.5204 (3)	0.0522 (10)	
H13A	−0.3205	0.1371	0.5821	0.063*	
H13B	−0.3971	0.2072	0.4854	0.063*	
C14	−0.3384 (6)	0.0684 (3)	0.4514 (3)	0.0581 (11)	
H14A	−0.4675	0.0465	0.4307	0.087*	
H14B	−0.2532	0.0156	0.4876	0.087*	
H14C	−0.3170	0.0862	0.3913	0.087*	

### Atomic displacement parameters (Å^2^)

**Table:**

	*U*^11^	*U*^22^	*U*^33^	*U*^12^	*U*^13^	*U*^23^
Cu1	0.0276 (2)	0.01543 (19)	0.0310 (2)	−0.00135 (14)	0.01221 (17)	0.00164 (14)
Cl1	0.0399 (4)	0.0189 (4)	0.0290 (4)	−0.0081 (3)	0.0116 (3)	0.0005 (3)
Cl2	0.0375 (4)	0.0188 (4)	0.0416 (4)	−0.0010 (3)	0.0214 (4)	−0.0044 (3)
N1	0.0276 (13)	0.0190 (12)	0.0258 (13)	−0.0006 (11)	0.0116 (11)	0.0015 (10)
N2	0.0259 (13)	0.0171 (12)	0.0258 (13)	−0.0004 (10)	0.0113 (10)	−0.0018 (10)
C1	0.0290 (16)	0.0277 (16)	0.0307 (17)	−0.0014 (13)	0.0127 (14)	0.0044 (13)
C2	0.0263 (16)	0.0390 (18)	0.0329 (18)	−0.0026 (15)	0.0114 (14)	0.0017 (14)
C3	0.0287 (17)	0.0326 (17)	0.0346 (18)	0.0042 (14)	0.0074 (14)	0.0065 (14)
C4	0.0360 (18)	0.0232 (15)	0.0378 (18)	0.0020 (15)	0.0094 (15)	0.0050 (14)
C5	0.0298 (16)	0.0205 (14)	0.0299 (16)	0.0022 (13)	0.0127 (13)	0.0014 (12)
C6	0.0322 (17)	0.0186 (14)	0.0345 (17)	0.0005 (13)	0.0127 (14)	0.0043 (13)
C7	0.0268 (15)	0.0233 (15)	0.0298 (16)	−0.0013 (13)	0.0126 (13)	0.0006 (12)
C8	0.0275 (18)	0.0250 (17)	0.069 (3)	0.0008 (15)	0.0081 (17)	0.0166 (17)
C9	0.0330 (19)	0.0299 (19)	0.082 (3)	−0.0036 (16)	0.009 (2)	0.0176 (19)
C10	0.0278 (17)	0.0346 (19)	0.047 (2)	−0.0026 (15)	0.0136 (15)	0.0002 (15)
C11	0.0265 (16)	0.0283 (16)	0.0328 (17)	0.0048 (13)	0.0090 (14)	−0.0013 (13)
C12	0.0300 (16)	0.0193 (14)	0.0317 (17)	0.0016 (13)	0.0097 (13)	0.0000 (12)
C13	0.033 (2)	0.042 (2)	0.076 (3)	−0.0026 (17)	0.0158 (19)	0.004 (2)
C14	0.048 (2)	0.072 (3)	0.048 (2)	−0.023 (2)	0.0130 (19)	−0.003 (2)

### Geometric parameters (Å, º)

**Table:**

Cu1—N2	2.037 (2)	C6—H6A	0.9500
Cu1—N1	2.079 (2)	C7—C12	1.379 (4)
Cu1—Cl2	2.2876 (8)	C7—C8	1.393 (4)
Cu1—Cl1	2.3067 (8)	C8—C9	1.375 (5)
Cu1—Cl1^i^	2.4321 (8)	C8—H8A	0.9500
Cl1—Cu1^i^	2.4321 (8)	C9—C10	1.389 (5)
N1—C1	1.328 (4)	C9—H9A	0.9500
N1—C5	1.350 (4)	C10—C11	1.382 (4)
N2—C6	1.280 (4)	C10—C13	1.523 (5)
N2—C7	1.437 (4)	C11—C12	1.388 (4)
C1—C2	1.390 (4)	C11—H11A	0.9500
C1—H1A	0.9500	C12—H12A	0.9500
C2—C3	1.374 (4)	C13—C14	1.520 (6)
C2—H2A	0.9500	C13—H13A	0.9900
C3—C4	1.384 (4)	C13—H13B	0.9900
C3—H3A	0.9500	C14—H14A	0.9800
C4—C5	1.383 (4)	C14—H14B	0.9800
C4—H4A	0.9500	C14—H14C	0.9800
C5—C6	1.453 (4)		
			
N2—Cu1—N1	80.94 (9)	N2—C6—H6A	119.9
N2—Cu1—Cl2	94.43 (7)	C5—C6—H6A	119.9
N1—Cu1—Cl2	119.40 (7)	C12—C7—C8	119.1 (3)
N2—Cu1—Cl1	171.55 (7)	C12—C7—N2	119.5 (3)
N1—Cu1—Cl1	93.80 (7)	C8—C7—N2	121.4 (3)
Cl2—Cu1—Cl1	93.91 (3)	C9—C8—C7	119.7 (3)
N2—Cu1—Cl1^i^	90.23 (7)	C9—C8—H8A	120.1
N1—Cu1—Cl1^i^	113.66 (7)	C7—C8—H8A	120.1
Cl2—Cu1—Cl1^i^	126.81 (3)	C8—C9—C10	122.0 (3)
Cl1—Cu1—Cl1^i^	85.82 (3)	C8—C9—H9A	119.0
Cu1—Cl1—Cu1^i^	94.18 (3)	C10—C9—H9A	119.0
C1—N1—C5	118.0 (3)	C11—C10—C9	117.5 (3)
C1—N1—Cu1	130.8 (2)	C11—C10—C13	121.8 (3)
C5—N1—Cu1	111.22 (19)	C9—C10—C13	120.7 (3)
C6—N2—C7	119.9 (2)	C10—C11—C12	121.3 (3)
C6—N2—Cu1	112.36 (19)	C10—C11—H11A	119.3
C7—N2—Cu1	127.69 (18)	C12—C11—H11A	119.3
N1—C1—C2	122.4 (3)	C7—C12—C11	120.3 (3)
N1—C1—H1A	118.8	C7—C12—H12A	119.9
C2—C1—H1A	118.8	C11—C12—H12A	119.9
C3—C2—C1	119.5 (3)	C14—C13—C10	113.4 (3)
C3—C2—H2A	120.3	C14—C13—H13A	108.9
C1—C2—H2A	120.3	C10—C13—H13A	108.9
C2—C3—C4	118.6 (3)	C14—C13—H13B	108.9
C2—C3—H3A	120.7	C10—C13—H13B	108.9
C4—C3—H3A	120.7	H13A—C13—H13B	107.7
C5—C4—C3	118.7 (3)	C13—C14—H14A	109.5
C5—C4—H4A	120.7	C13—C14—H14B	109.5
C3—C4—H4A	120.7	H14A—C14—H14B	109.5
N1—C5—C4	122.8 (3)	C13—C14—H14C	109.5
N1—C5—C6	115.0 (3)	H14A—C14—H14C	109.5
C4—C5—C6	122.2 (3)	H14B—C14—H14C	109.5
N2—C6—C5	120.3 (3)		
			
N2—Cu1—Cl1—Cu1^i^	62.3 (5)	Cu1—N1—C5—C4	−179.1 (2)
N1—Cu1—Cl1—Cu1^i^	113.48 (7)	C1—N1—C5—C6	−179.5 (3)
Cl2—Cu1—Cl1—Cu1^i^	−126.67 (3)	Cu1—N1—C5—C6	2.2 (3)
Cl1^i^—Cu1—Cl1—Cu1^i^	0.0	C3—C4—C5—N1	1.7 (5)
N2—Cu1—N1—C1	178.7 (3)	C3—C4—C5—C6	−179.7 (3)
Cl2—Cu1—N1—C1	−91.4 (3)	C7—N2—C6—C5	176.6 (3)
Cl1—Cu1—N1—C1	5.3 (3)	Cu1—N2—C6—C5	−4.4 (4)
Cl1^i^—Cu1—N1—C1	92.4 (3)	N1—C5—C6—N2	1.5 (4)
N2—Cu1—N1—C5	−3.39 (19)	C4—C5—C6—N2	−177.3 (3)
Cl2—Cu1—N1—C5	86.6 (2)	C6—N2—C7—C12	168.0 (3)
Cl1—Cu1—N1—C5	−176.73 (19)	Cu1—N2—C7—C12	−10.8 (4)
Cl1^i^—Cu1—N1—C5	−89.63 (19)	C6—N2—C7—C8	−12.8 (4)
N1—Cu1—N2—C6	4.2 (2)	Cu1—N2—C7—C8	168.4 (3)
Cl2—Cu1—N2—C6	−114.9 (2)	C12—C7—C8—C9	−2.7 (5)
Cl1—Cu1—N2—C6	56.1 (6)	N2—C7—C8—C9	178.1 (3)
Cl1^i^—Cu1—N2—C6	118.1 (2)	C7—C8—C9—C10	0.1 (6)
N1—Cu1—N2—C7	−176.9 (2)	C8—C9—C10—C11	2.1 (6)
Cl2—Cu1—N2—C7	64.0 (2)	C8—C9—C10—C13	−178.3 (4)
Cl1—Cu1—N2—C7	−125.0 (4)	C9—C10—C11—C12	−1.8 (5)
Cl1^i^—Cu1—N2—C7	−63.0 (2)	C13—C10—C11—C12	178.6 (3)
C5—N1—C1—C2	−0.4 (4)	C8—C7—C12—C11	2.9 (5)
Cu1—N1—C1—C2	177.5 (2)	N2—C7—C12—C11	−177.8 (3)
N1—C1—C2—C3	0.6 (5)	C10—C11—C12—C7	−0.7 (5)
C1—C2—C3—C4	0.3 (5)	C11—C10—C13—C14	123.0 (4)
C2—C3—C4—C5	−1.4 (5)	C9—C10—C13—C14	−56.6 (5)
C1—N1—C5—C4	−0.8 (4)		

Symmetry code: (i) −*x*+1, −*y*+1, −*z*+1.

### Hydrogen-bond geometry (Å, º)

**Table:**

*D*—H···*A*	*D*—H	H···*A*	*D*···*A*	*D*—H···*A*
C1—H1*A*···Cl1	0.95	2.80	3.364 (3)	119
C2—H2*A*···Cl2^ii^	0.95	2.76	3.445 (3)	130
C6—H6*A*···Cl2^iii^	0.95	2.62	3.506 (3)	155
C12—H12*A*···Cl2	0.95	2.80	3.450 (3)	126

Symmetry codes: (ii) *x*+1, *y*, *z*; (iii) −*x*+1, *y*−1/2, −*z*+3/2.
